# Investigating UV-Irradiation Parameters in the Green Synthesis of Silver Nanoparticles from Water Hyacinth Leaf Extract: Optimization for Future Sensor Applications

**DOI:** 10.3390/nano14121018

**Published:** 2024-06-12

**Authors:** Fueangfakan Chutrakulwong, Kheamrutai Thamaphat, Mana Intarasawang

**Affiliations:** 1 Division of Physics, Faculty of Science and Technology, Rajamangala University of Technology Krungthep, Bangkok 10120, Thailand; fueangfakan.c@mail.rmutk.ac.th; 2 Green Synthesis and Application Laboratory, Applied Science and Engineering for Social Solution Research Unit, Department of Physics, Faculty of Science, King Mongkut’s University of Technology Thonburi, Bangkok 10140, Thailand; 3 Department of Science and Technology, Suksanari School, Bangkok 10600, Thailand; mana_in@snr.ac.th

**Keywords:** green synthesis, silver nanoparticles, surface plasmon resonance, UV irradiation, water hyacinth

## Abstract

Silver nanoparticles (AgNPs) can be produced safely and greenly using water hyacinth, an invasive aquatic plant, as a reducing agent. This study aimed to optimize the UV-irradiation parameters for the synthesis of AgNPs from water hyacinth leaf extract. The study varied the reaction time and pH levels and added a stabilizing agent to the mixture. The synthesized AgNPs were characterized using UV-visible spectroscopy (UV-vis), transmission electron microscopy (TEM), X-ray diffraction (XRD), Fourier transform infrared spectroscopy (FTIR), and inductively coupled plasma optical emission spectroscopy (ICP-OES). The findings revealed that the optimal conditions for synthesizing AgNPs were achieved by adjusting the pH level to 8.5, adding starch as a stabilizing agent, and exposing the mixture to UV-A radiation for one hour. These conditions resulted in the smallest size and highest quantity of AgNPs. Furthermore, the synthesized AgNP colloids remained stable for up to six months. This study highlights the potential of utilizing water hyacinth as a sustainable and cost-effective reducing agent for AgNP synthesis, with potential applications in pharmaceuticals, drug development, catalysis, and sensing detection.

## 1. Introduction

In recent years, nanoparticles have obtained remarkable attention due to their exclusive properties and potential utilization [1] in various fields, including medicine [2], cosmetics [3], textiles [4], engineering, catalysis [5], and environmental remediation [6,7]. Among the various types of nanoparticles, silver nanoparticles (AgNPs) have been extensively investigated because of their excellent optical [8], electrical [9], and biological properties [10,11,12,13]. However, the traditional methods for AgNP synthesis use toxic chemicals [10,14] and solvents, which pose a significant threat to the environment and human health [14,15,16].

AgNPs can synthesized using physical, chemical, and biological approaches [10,17]. The first two methods are expensive and require high energy inputs, temperatures, and pressures [10]. Moreover, the chemicals used for nanoparticle synthesis and stabilization are toxic and lead to non-ecofriendly by-products [18]. In contrast, the biological method [19] using microorganisms [20], algae [21,22], or plant extracts [23,24,25,26] to generate nanoparticles in the same way that they are produced in nature is much cleaner and is friendly to the environment. However, synthesis via plant-based methods is faster and easier than microorganism-based synthesis because it doesn’t contain the risk of microbial contamination [20,27,28]. Therefore, an interest in preparing AgNPs using the green synthesis method has been increasing in recent years [12,29,30,31,32,33,34,35].

Recent research has explored the synthesis of AgNPs using a wide variety of plant parts, leveraging their inherent reducing and stabilizing properties. Various studies have demonstrated the successful synthesis of AgNPs using extracts from the leaves, stems, roots, and fruits of numerous plants. For instance, extracts from the leaves of *Azadirachta indica* (neem) [36] and *Mangifera indica* (mango) [37] have been utilized for their high content of reducing sugars and polyphenols, which facilitate the reduction of silver ions to AgNPs. Similarly, root extracts of *Beta vulgaris* (beetroot) [38] and fruit extracts of *Musa paradisiaca* (banana) [39] have been employed, demonstrating the versatility of different plant parts in the green synthesis of nanoparticles.

The term ‘green’ highlights the utilization of plant-based materials in the production of nanoparticles. In this context, water hyacinths (*Eichhornia crassipes*) present a unique opportunity. Known for their rapid growth and invasive nature, water hyacinths pose significant environmental challenges by clogging waterways and depleting oxygen levels in aquatic ecosystems [40,41]. Utilizing water hyacinth biomass for the synthesis of AgNPs not only provides a sustainable and eco-friendly method for nanoparticle production but also offers a potential solution to mitigate the environmental pollution caused by this invasive species [42,43,44,45,46,47]. The abundant polyphenols and flavonoids present in water hyacinths can act as both reducing and stabilizing agents, making them an ideal candidate for green synthesis [48]. This approach not only addresses the environmental impact of water hyacinths but also contributes to the development of cost-effective and sustainable methods for producing AgNPs with significant antimicrobial properties.

Our work is not the first to use water hyacinth to synthesize AgNPs. Many researchers have previously used water hyacinth for this purpose [42,43,49,50]. However, their methods differ from ours. Other researchers used heating as a catalyst in the AgNP synthesis process, which takes several hours to form nanoparticles with sufficient antibacterial properties, typically between 3–50 h. Additionally, the synthesized AgNPs in their studies have a large size range of 16–240 nm [42,43,49,50]. The size-dependent properties of AgNPs influence their effectiveness, with smaller nanoparticles generally being more effective due to their higher surface area and reactivity. Among these, spherical nanoparticles with the smallest size exhibit the strongest antibacterial action compared to triangular and larger spherical shapes [51,52]. For excellent antibacterial properties, AgNPs should have a spherical shape and an average diameter in the range of 1–10 nm, as these provide the best antibacterial efficiency and can directly destroy cell surfaces [53,54].

To address these challenges, we chose to study water hyacinth in depth and improve the upscaling process to produce AgNPs more efficiently. Our synthesis process is shorter and faster, and the AgNPs can be applied in medicine, as antibacterials, and as sensors. Moreover, no other research has demonstrated that the shelf life of AgNPs can be longer, more effective, or more stable than our findings.

This research aimed to study the synthesis of AgNPs from water hyacinth extract using UV irradiation because specific wavelengths of light can better control the size and shape of AgNPs compared to the heating method. The energy from light can be directed to a specific wavelength, aiding in controlling and uniformly reducing the process. Consequently, the narrower the size distribution of nanoparticles, the smaller they will be. Additionally, the effect of reaction time was investigated to determine the fastest conditions for synthesis. Furthermore, no other research has explored the effect of pH, which significantly influences the size, shape, quantity, and stability of AgNPs. Thus, this research examined different types of UV irradiation, reaction times, the effect of pH on AgNPs, and the shelf life of AgNPs to maximize efficiency and stability.

Our study demonstrates that the AgNPs synthesized using our technique exhibit exceptional long-term stability. Rigorous testing has confirmed that these nanoparticles maintain their physicochemical properties and antimicrobial efficacy even after six months of storage. This prolonged stability is attributed to the effective stabilization provided by the natural reducing agents and stabilizers derived from water hyacinth plants. The consistent performance over an extended period underscores the robustness of our synthesis method and its potential for practical applications in various fields, including the medical and environmental sectors.

## 2. Materials and Methods

**Figure 1:**
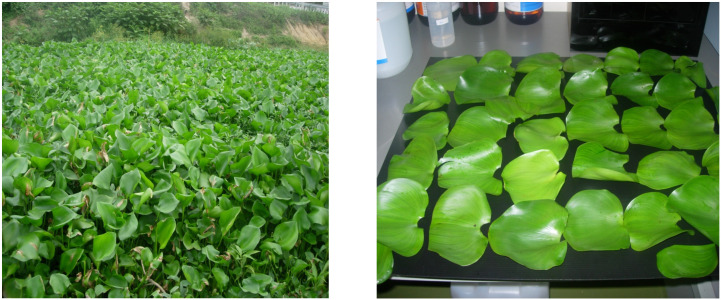
Water hyacinth plant photographs from a drainage canal in Lak Song, Bang Khae, Bangkok, Thailand.

The water hyacinth plant (Figure 1), collected from a drainage canal in Lak Song, Bang Khae, Bangkok, Thailand, was used throughout the experiment. The materials used in this study included silver nitrate (AgNO_3_) (99.9% purity, Sigma-Aldrich, St. Louis, MO, USA), water hyacinth leaves (collected from a canal in Bangkok, Thailand), sodium hydroxide (NaOH) (99.9% purity, Sigma-Aldrich), and starch (Sigma-Aldrich).

### 2.1. Green Synthesis of AgNPs

The green synthesis of AgNPs was meticulously carried out using water hyacinth leaf extract as a reducing agent. The extract was prepared by washing the leaves with distilled water and drying them in the surrounding circumstances for 24 h. Then, the dried leaves were ground into a fine powder and mixed with deionized water at 1:10 (*w*/*v*). The mixture was heated at 100 °C for 30 min and filtered through a Whatman filter paper to obtain the water hyacinth leaf extract.

To synthesize AgNPs, a 10 mM AgNO_3_ solution was prepared by dissolving AgNO_3_ in deionized water. This solution was mixed with the water hyacinth leaf extract at a ratio of 1:1 (*v*/*v*). The mixture was stirred for 1 h at room temperature to allow for the reduction of Ag^+^ ions to Ag^0^. The mixture was exposed to UV-A radiation (365 nm) for one hour to speed up the reaction.

### 2.2. Optimization of Conditions for Synthesized AgNPs

To optimize the UV-irradiation parameters for the green synthesis of AgNPs, the reaction time, pH levels, and addition of a stabilizing agent were varied. The reaction time was varied from 5 to 60 min, and the pH levels were adjusted using NaOH and HCl solutions. The addition of starch as a stabilizing agent was also tested.

### 2.3. Characterization of AgNPs

The synthesized AgNPs were characterized using UV-vis, TEM, XRD, FTIR, and ICP-OES. UV-vis was used to determine the absorbance spectra of the synthesized AgNPs, and TEM was used to determine the size and morphology of the particles. XRD was used to determine the crystalline structure of the generated AgNPs. FTIR was employed to comprehend the AgNP synthesis mechanism. ICP-OES was used to determine the concentration of Ag in the synthesized AgNPs.

### 2.4. Statistical Analysis

The statistics used to describe the finding included mean ± standard deviation, one-way analysis of variance (ANOVA), and Tukey’s post-hoc test. The hypothesis test is considered statistically significant when the *p*-value is less than 0.05.

## 3. Results and Discussion

### 3.1. Effect of UV-Irradiation Parameters on AgNP Synthesis

**Figure 2:**
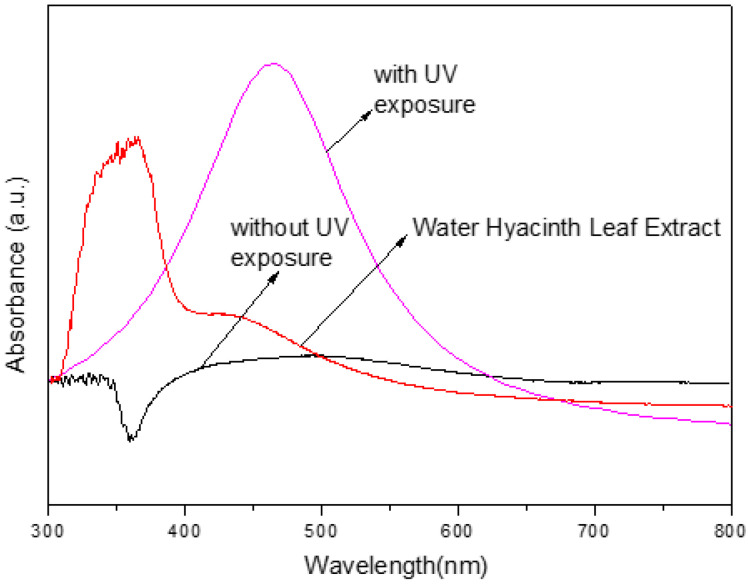
Spectrum of the UV light absorption of sample solutions with and without UV exposure for 1 h.

Figure 2 shows the UV-vis spectra of the sample solutions with and without UV exposure. The *x*-axis represents the wavelength of light in nanometers (nm), and the *y*-axis represents the absorbance intensity. The red line represents the sample solution containing only water hyacinth leaf extract, which was used as a blank for comparison. The black line represents the sample solution containing water hyacinth leaf extract and AgNO_3_ solution without pH adjustment and UV exposure. The pink line represents the sample solution containing water hyacinth leaf extract and AgNO_3_ solution without pH adjustment but with UV exposure.

The figure shows that the sample solution with UV exposure has a higher absorbance intensity than the solution without, indicating that UV radiation helped increase the quantity of AgNPs synthesized. The absorbance peak at around 430 nm in the pink line indicates the presence of AgNPs in the sample solution. The blank sample solution (red line) shows no significant absorbance peak, indicating the absence of AgNPs.

**Figure 3:**
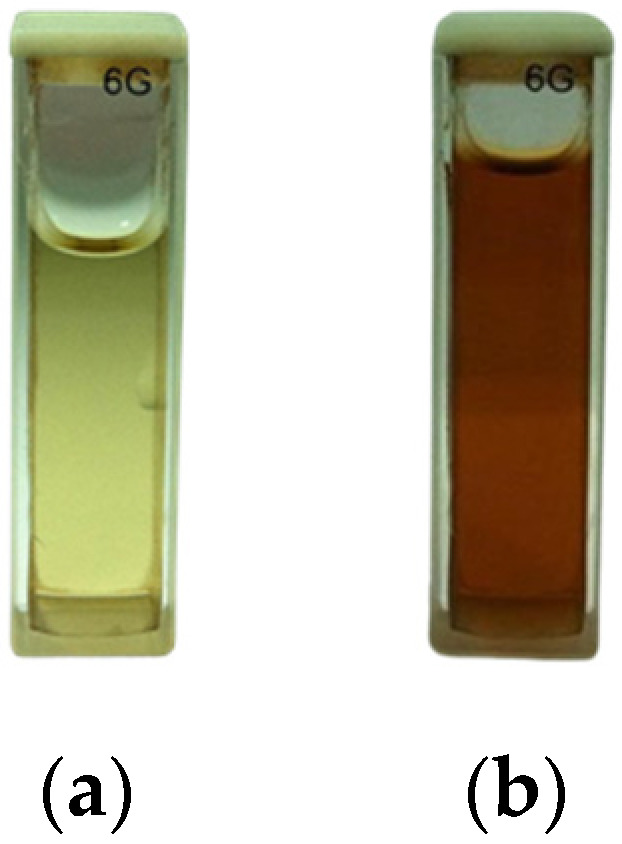
Colors of sample mixtures at the baseline: (**a**) Water hyacinth leaf extract and (**b**) after 1 h of exposure to UV radiation.

Figure 3 shows the color change of the sample solution before and after UV radiation exposure. Panel (a) shows the color of the mixed solution before the reaction, which is light yellow. Panel (b) shows the color of the AgNPs colloid after 1 h of exposure to UV radiation, which is dark brown.

**Figure 4:**
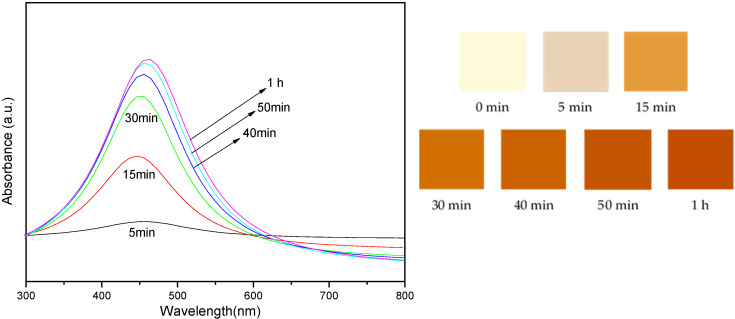
Images showing the color of the colloidal AgNP suspensions after exposure to UV-A light for varying reaction times and the absorbance intensity of the sample mixture were exposed to UV-A light under a variation of reaction times.

The formation of AgNPs in the reaction mixture is reflected in the color change of the mixture from light yellow to dark brown. This outcome was due to the surface plasmon resonance (SPR) effect, a metallic nanoparticle characteristic. The SPR effect occurs when the electrons on the surface of the nanoparticles are excited by incident light, causing a collective oscillation of the electrons and resulting in light absorption at a specific wavelength. In the case of AgNPs, the SPR effect occurs at around 430 nm, consistent with the UV-vis spectra shown in Figure 4.

The change in color, from the initial light yellow to the final dark brown, serves as a powerful visual confirmation of our successful synthesis of AgNPs. This transformation, brought about by the water hyacinth leaf extract acting as a reducing agent, signifies the presence of a high concentration of AgNPs in the reaction mixture, a significant step forward in our research.

### 3.2. Effect of Reaction Time on Different Types of UV Rays

Our investigation into the impact of reaction time on AgNP synthesis, spanning from 5 to 60 min, yielded intriguing results. We observed that the absorbance intensity of the synthesized AgNPs increased with longer reaction times, indicating a corresponding increase in the quantity of AgNPs. However, the size of the AgNPs did not exhibit a significant change, underscoring the complex nature of this synthesis process and the importance of reaction time as a variable. 

Figure 4 presents the absorbance intensity of the sample mixture exposed to UV-A light at different reaction times. The spectra are shown for reactions ranging from 5 min to 1 h.

The absorption spectra provide information about the formation and characteristics of the AgNPs in the reaction mixture. The presence of a peak in the spectra indicates the presence of AgNPs, and the position and intensity of the peak provide insights into the size and concentration of the nanoparticles.

Figure 4 shows the images of the color changes of colloidal AgNP suspensions after exposure to UV-A light for varying reaction times. The images depict the visual color of the suspensions at different reaction times: 0 min, 5 min, 15 min, 30 min, 40 min, 50 min, and 1 h. The graph presents the absorbance intensity of the sample mixtures corresponding to these reaction times, measured in the 300–800 nm wavelength range. The colors in the absorbance spectra correlate with the reaction times, with light yellow representing 0 min, brown representing 15 min, and so on.

Strong evidence for the presence of AgNPs was provided by the color of the colloidal AgNP suspensions shown in Figure 4, which displayed comparable UV-vis absorption spectra with an SPR peak at a wavelength of about 430 nm. When the irradiation period was prolonged, the absorbance intensity increased. This was mostly due to the production of additional particles with almost identical particle diameter sizes, which corresponded to the TEM results. These changes in color and absorbance intensity indicate nanoparticle formation, size variation, or concentration changes.

**Figure 5:**
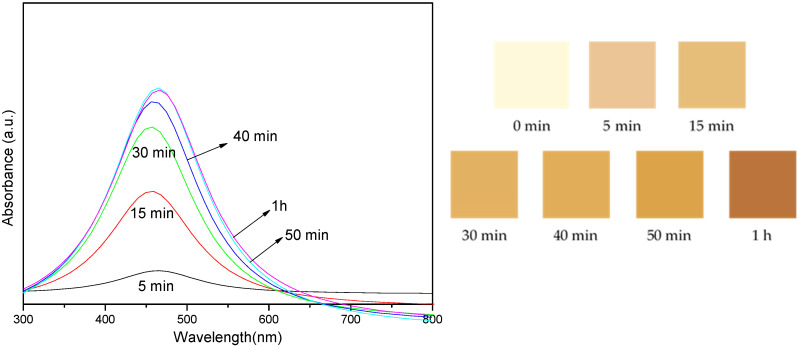
Images showing the color of the colloidal AgNP suspensions after exposure to UV-B light for varying reaction times and the absorbance intensity of the sample mixture exposed to UV-B light under a variation of reaction times.

Figure 5 shows the absorbance intensity of the sample mixtures exposed to UV-B radiation under variable reaction times. The spectra are shown for reactions ranging from 5 min to 1 h.

In Figure 5, the spectra show a similar trend to those in Figure 4, with an increase in absorbance intensity and a shift in the peak position towards a specific wavelength as the reaction progresses. The color changes in colloidal AgNP solutions exposed to UV-B light for various reaction times. The pictures show the suspensions’ visual colors at various reaction times, including 0 min, 5 min, 15 min, 30 min, 40 min, 50 min, and 1 h. The graph shows the sample mixtures’ absorbance intensity, measured in the 300–800 nm wavelength range, in relation to these reaction periods. The absorbance spectra’s hues correspond to the reaction timings: pale yellow denotes 0 min; brown, 15 min; and so forth. 

The color of the colloidal AgNP suspensions in Figure 5 demonstrated similar UV-vis absorption spectra with an SPR peak at around 440 nm, providing compelling evidence for the existence of AgNPs. The absorbance intensity rose as the irradiation period was extended. This increase was largely caused by the generation of additional particles, which matched the TEM results in terms of particle diameter sizes. However, the spectra for UV-B exposure show a slightly lower absorbance intensity than UV-A exposure, indicating that UV-A radiation is more effective in synthesizing AgNPs from the water hyacinth leaf extract.

**Figure 6:**
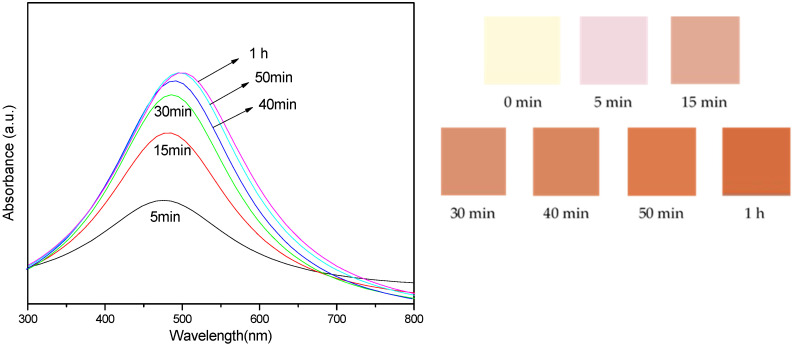
Images showing the color of the colloidal AgNP suspensions after exposure to UV-C light for varying reaction times and the absorbance intensity of the sample mixture exposed to UV-C light with variations in reaction times.

Figure 6 shows the mixture’s absorbance intensity after exposure to UV-C light at various reaction times. The spectra are shown for reactions ranging from 5 min to 1 h.

Figure 6, in line with Figure 4 and Figure 5, demonstrates a consistent trend. As the reaction progresses, there is a noticeable increase in absorbance intensity and a shift in the peak position towards a specific wavelength. The color alterations of colloidal AgNP solutions after varying reaction durations under UV-C light. The images display the colors of the suspensions at different reaction times: 0 min, 5 min, 15 min, 30 min, 40 min, 50 min, and 1 h. The graph displays the absorbance intensity of the sample mixtures in relation to these reaction times, recorded in the wavelength range of 300–800 nm. The colors of the absorbance spectra represent the reaction timings: light yellow indicates 0 min, brown indicates 15 min, and so on. 

Strong evidence for the presence of AgNPs was shown by the color of the colloidal AgNP suspensions in Figure 6, which displayed comparable UV-vis absorption spectra with an SPR peak at about 500 nm. As the irradiation period was prolonged, the absorption intensity increased. The production of additional particles, which matched the TEM data in terms of particle diameter sizes, was primarily responsible for this increase. Notably, the spectra for UV-C exposure exhibit the highest absorbance intensity, surpassing that of UV-A and UV-B light. This underscores the superior efficiency of UV-C light in synthesizing AgNPs from water hyacinth leaf extract. However, it is worth noting that the spectra for UV-C exposure also reveal a broader peak, suggesting a wider size distribution of the synthesized AgNPs. This implies that UV-C radiation may lead to larger AgNPs’ size and dispersion compared to UV-A and UV-B radiation.

For all of the above, a UV-vis spectrophotometer was used to track the AgNP production process. The recorded solution color and absorption spectra of AgNPs, as shown in Figure 4, Figure 5 and Figure 6, indicate that the absorbance intensity at λ_max_ increased as exposure time increased. Additionally, a redshift in the absorbance peak’s wavelength to a longer wavelength was noted under different UV light conditions (UV-A, UV-B, and UV-C, respectively). Consequently, both AgNPs and other metal nanoparticles are synthesized. Initially, the UV-vis spectrophotometer can be used to measure the size and distribution of the particles, which is confirmed with a TEM to double-check the size of the AgNPs, as discussed in the following section.

### 3.3. Characterization of Green Synthesized AgNPs

**Figure 7:**
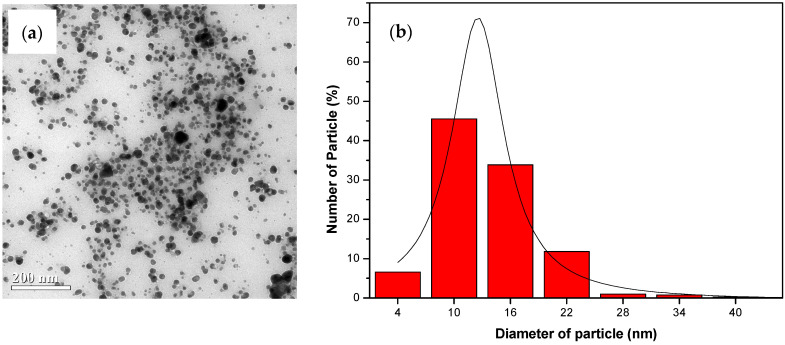
The analysis results from TEM: (**a**) The image of the colloid AgNPs generated from water hyacinth leaf extract under 1 h of UV-A light exposure; (**b**) a histogram of corresponding AgNP particle size distribution.

**Figure 8:**
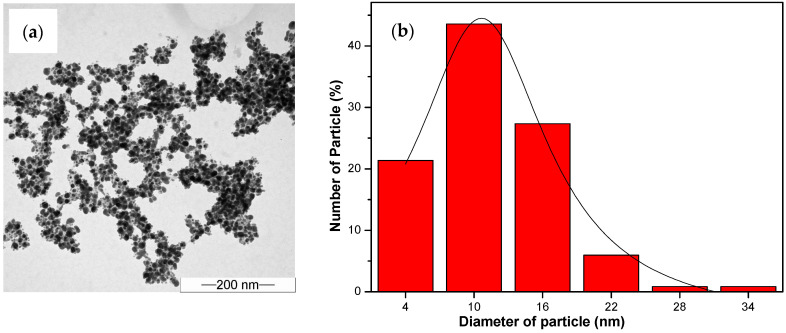
The analysis results from TEM: (**a**) The image of the colloid AgNPs generated from water hyacinth leaf extract under 1 h of UV-B light exposure; (**b**) a histogram of corresponding AgNP particle size distribution.

**Figure 9:**
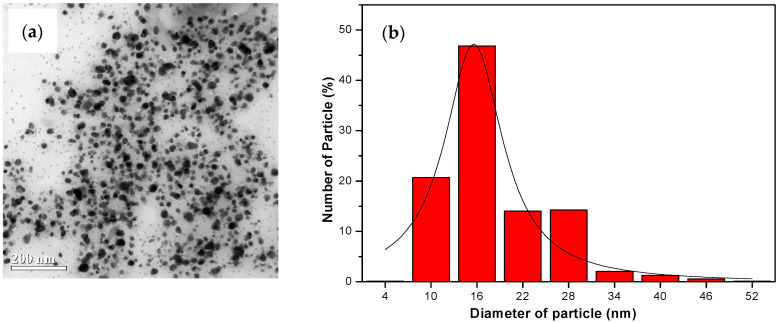
The analysis results from TEM: (**a**) The image of the colloid AgNPs generated from water hyacinth leaf extract under 1 h of UV-C light exposure; (**b**) a histogram of corresponding AgNP particle size distribution.

The TEM image (Figure 7a, Figure 8a and Figure 9a) provides visual insight into the morphology and dispersion of the synthesized AgNPs. The image reveals the presence of sphere-shaped nanoparticles, indicating that the synthesis process resulted in the formation of relatively uniform nanoparticles.

The corresponding particle size distribution histogram (Figure 7b, Figure 8b and Figure 9b) provides quantitative information about the size distribution of the AgNPs. By analyzing the histogram, it is possible to determine the average size of the nanoparticles and assess the uniformity of the size distribution. This information is valuable for understanding the effectiveness of the synthesis process and for controlling the size of the nanoparticles for specific applications.

The results from the TEM analysis suggest that the synthesis of AgNPs from water hyacinth leaf extract with exposure to UV irradiation (UV-A et al.) for 1 h resulted in the formation of relatively uniform and small nanoparticles, as indicated by the TEM image and the particle size distribution histogram. This finding is consistent with the previous results indicating that UV-A, UV-B, and UV-C irradiation and specific reaction conditions contribute to synthesizing AgNPs with desired characteristics.

Table 1 presents the λ_max_, FWHM, and average sizes of AgNPs synthesized from water hyacinth leaf extract exposed to different types of UV radiations, namely UV-A, UV-B, and UV-C. 

λ_max_ (nm) refers to the wavelength at which the maximum absorbance of the synthesized AgNPs occurs. A statistical metric called full width at half maximum (FWHM) is used to characterize the width of a Gaussian or normal distribution. In particular, it is the breadth of a curve, measured between the two points where the curve’s height is half of its maximum value. The average size of the AgNPs is also provided in nanometers (nm).

The results in Table 1 indicate that the AgNPs synthesized under UV-A irradiation had the maximum absorption wavelength (λ_max_ = 430 nm) and the smallest average size (12.54 ± 0.19 nm), and the FWHM was measured to be 137.73 nm. This suggests that UV-A irradiation produces small and uniformly sized AgNPs most effectively. 

On the other hand, the AgNPs synthesized under UV-C irradiation had the maximum absorption wavelength (λ_max_ = 500 nm) and the largest average size (18.39 ± 0.48 nm), and the FWHM was measured to be 173.54 nm. This indicates that UV-C irradiation produces small and uniformly sized AgNPs less effectively.

The effects of different types of UV radiation (UV-A, UV-B, and UV-C) on the synthesis of AgNPs were studied using water hyacinth leaf extract. The study found that when AgNPs were synthesized under UV-A irradiation conditions, the absorption intensity at a wavelength of around 430 nm was higher. This indicated an increased AgNP formation rate, suggesting that a greater amount of AgNPs could be synthesized. Additionally, the synthesized AgNPs were smaller and more flexible, with a small size distribution observed from the relatively low full width at half maximum (FWHM) value. The synthesized particles had a diameter of 12.54 ± 0.19 nm.

In comparison, AgNPs synthesized under UV-B and UV-C conditions showed absorption intensities at wavelengths around 440 nm and 500 nm, respectively. The synthesized AgNPs had diameters of 13.14 ± 0.23 nm and 18.39 ± 0.48 nm, respectively. Therefore, in this work, UV-A radiation was chosen as the catalyst for the synthesis of AgNPs because it produced the smallest AgNPs compared to UV-B and UV-C radiation.

In the next section, the effect of the pH of the solution will be studied under appropriate conditions for the synthesis of AgNPs, with UV-A as the catalyst. The pH of the solution will also affect the rate of formation, size, and size distribution of AgNPs. When the solution is alkaline, the AgNP formation rate increases and the synthesized AgNPs are small, with a narrow size distribution. The optimum solution pH for the method presented in this work is 8.5. Under these conditions, the synthesized particles are spherical with an average diameter of 10.60 ± 0.19 nm, and the yield of synthesized AgNPs is as high as 99.87%.

**Figure 10:**
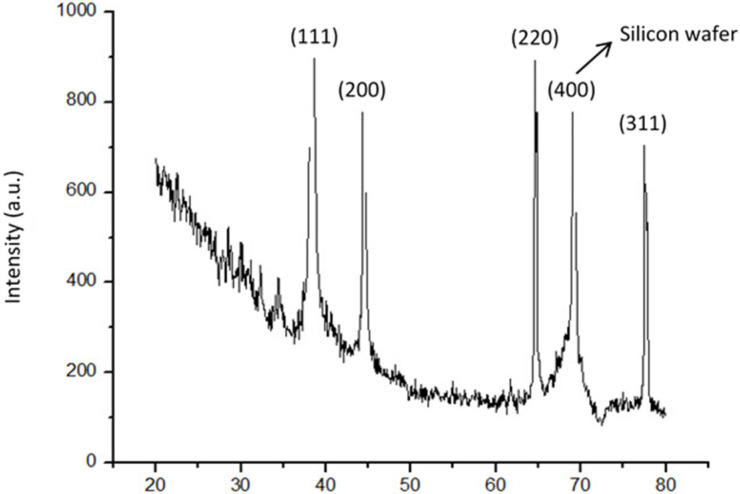
X-ray diffraction pattern of synthesized AgNPs.

XRD was used to characterize the produced AgNPs further. Figure 10 displays the XRD pattern. With diffraction peaks at 38.34°, 44.51°, 64.65°, and 77.58° in the 2θ range of 20° to 80°, it displays the distinct peaks of the face-centered cubic (fcc) crystal structure (JCPDS 04-0783), which correspond to the (111), (200), (220), and (311) facets of silver, respectively.

**Figure 11:**
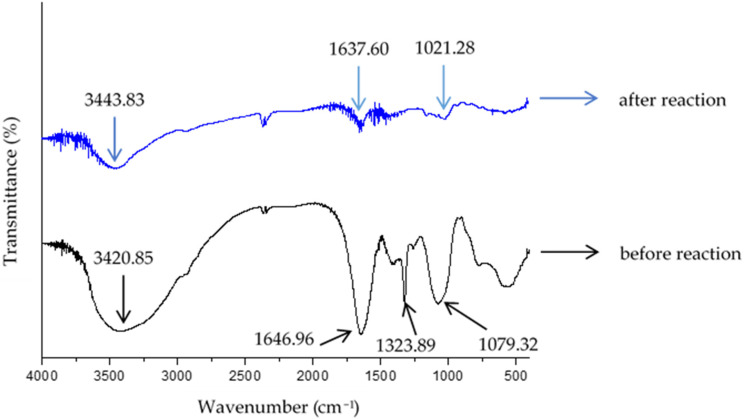
FITR spectrum of water hyacinth leaf extract before and after reactions with AgNO_3_.

Figure 11 displays the FTIR spectrum of the water hyacinth leaf extract produced in this investigation both before and after interactions with AgNO_3_. Prominent absorption bands at 1021.28 cm^−1^, 1637.60 cm^−1^, and 3443.83 cm^−1^ were displayed by the AgNPs. The band’s distance from the C-O stretching ether is approximately 1021.28 cm^−1^. It is suggested that phenolic compounds are connected to silver nanoparticles by a band at 3443.83 cm^−1^, which can be assigned to OH-stretching vibrations, and a band at 1637.60 cm^−1^, allocated to OH-bending vibrations [43]. Water hyacinth’s phenolic components, which include tannins, flavonoids, and other polyphenolic substances, function as reducing agents during the AgNP synthesis process [48]. However, after the process was finished, it was evident that all of the FTIR peaks’ transmission intensities decreased.

**Figure 12:**
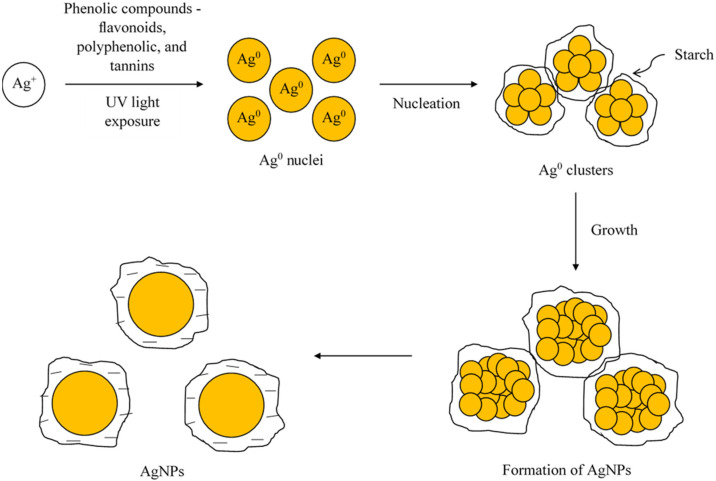
Schematic diagram of AgNP formation using water hyacinth leaf extract and AgNO_3_ solution.

These organic compounds contain one or more phenol functional groups (OH attached to an aromatic ring). Phenolic compounds, including flavonoids, tannins, and other polyphenolic compounds found in water hyacinth, demonstrate their unique chemical properties by serving as crucial reducing agents in the synthesis process. These compounds play a pivotal role by donating electrons from silver ions (Ag^+^) to silver atoms (Ag^0^), thereby facilitating their reduction to silver nanoparticles (AgNPs). By applying UV light irradiation to speed up the synthetic reaction, the starch that predominated in the extract worked as an excellent particle-stabilizing agent in the creation of AgNPs dispersion with high stability, as depicted in Figure 12. 

**Figure 13:**
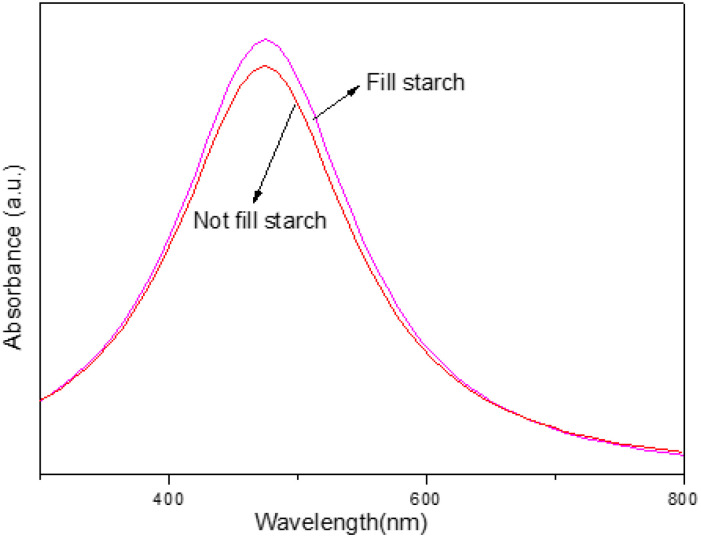
Absorbance spectra of AgNP solution with fill starch and no fill starch.

The effect on the absorbance of the synthesized AgNPs of adding a stabilizer (fill starch) was investigated. The sample solutions were prepared with and without fill starch, and both were exposed to UV-A for 1 h. The absorbance of the AgNPs was then examined using UV-vis, and the results are presented in Figure 13.

The absorbance spectra in Figure 13 show that the sample solution with fill starch had a higher intensity of AgNPs colloids than the one without fill starch. This result indicates that adding a stabilizer (starch) increased the quantity of AgNPs synthesized from water hyacinth leaf extract. 

Stabilizers are commonly used in nanoparticle synthesis to prevent aggregation and improve the stability of the nanoparticles. In this case, adding fill starch as a stabilizer likely prevented the AgNPs from aggregating and improved their stability, resulting in a higher quantity of synthesized AgNPs.

### 3.4. Effect of pH Levels

**Figure 14:**
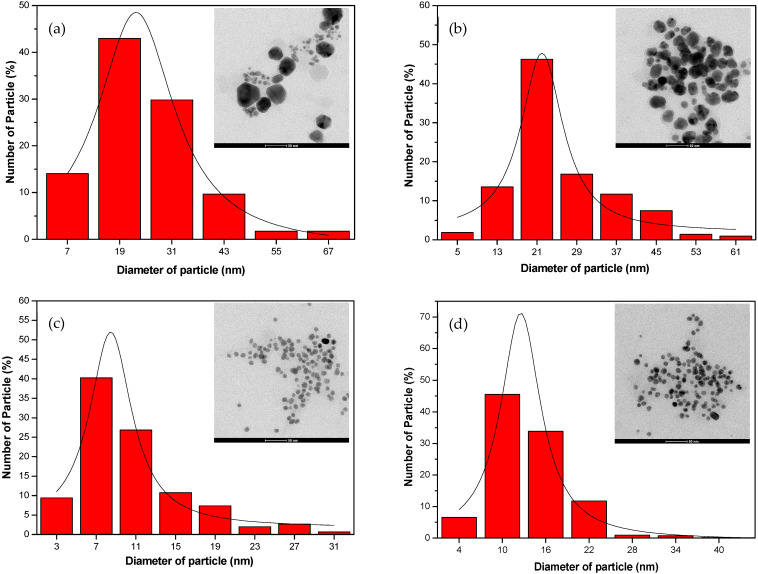
Representative TEM images and histogram of size distribution of as-prepared AgNPs produced under UV-A irradiation for 1 h: (**a**) pH 4.5; (**b**) pH 5.4; (**c**) pH 8.5; (**d**) pH 12.

In this section, we will examine how pH affects AgNPs’ size by observing size changes brought on by solution pH changes. The findings of all the TEM measurements of the produced samples at various pH values are shown in Figure 14. It is evident that the particle sizes drop and their shapes become more spherical as the pH value rises. Notably, there is a strong correlation between the absorption spectra of the identical samples and the TEM imaging results. The majority of the particles produced at low pH levels (4.5 and 5.4) had irregular shapes, as shown in Figure 14. In addition to the unbalanced nucleation and growth processes, irregularities in the morphology of the particles can be ascribed to the delayed rate of precursor reduction. Conversely, the AgNPs produced at high pH (8.5 and 12) are smaller and more regular than the earlier samples produced at lower pH levels. AgNPs have a spherical form with an average radius of around (10.60 ± 0.19 and 12.56 ± 0.42) nm. The equilibrium between the nucleation and growth processes as well as the increased rate of reduction of the silver precursor (Ag^+^) are responsible for the spherical form of AgNPs.

Additionally, the addition of starch as a stabilizer for synthesized AgNPs is highly dependent on the pH of the reaction mixture. At a neutral to slightly alkaline pH, starch effectively prevents agglomeration and stabilizes the size and shape of the nanoparticles. Low-pH conditions reduce the stabilizing efficiency of starch, leading to more agglomeration and less uniform nanoparticles. High-pH conditions, while promoting rapid nucleation, still benefit from the strong stabilizing effect of starch, resulting in stable and well-dispersed nanoparticles.

Our investigation involved adjusting the pH levels of the reaction mixture using NaOH and HCl solutions to study the effect on AgNP synthesis. Interestingly, the pH level of 8.5 produced the most major and significant quantities of AgNPs.

The sample mixtures were prepared with different pH levels ranging from 4.5 to 12, and the concentration of Ag in the synthesized AgNPs was measured using ICP-OES.

The outcome indicates that the mixture solution’s pH level significantly impacts the quantity and size of the synthesized AgNPs. As shown in Table 2, the sample solution with a pH level of 8.5 generated the smallest size of AgNPs (10.60 ± 0.19 nm) with the highest quantity (99.87% yield). Meanwhile, the solution with a pH of 12 provided a relatively small size (12.56 ± 0.42 nm) and a high quantity of AgNPs (96.07% yield), close to the one with pH 8.5. Therefore, pH 8.5 was recommended in AgNPs synthesized from water hyacinth extract because the pH value could be adjusted more easily than pH 12. 

The finding indicates that the pH level of the reaction mixture significantly affects the size and quantity of the synthesized AgNPs because the pH level affects the reduction potential of the reducing agents in the extract, which in turn affects the silver ions’ reduction rate and the size of the AgNPs. At higher pH levels, the reducing agents are more effective in reducing the silver ions, resulting in smaller and more uniform AgNPs.

The size of colloidal AgNPs determines their λ_max_ value, with λ_max_ shifting toward longer wavelengths as AgNPs grow bigger. The results in Table 2 demonstrated that the λ_max_ of AgNPs corresponded to particle sizes of 25.25 nm, 24.74 nm, 10.60 nm, and 12.56 nm at pH 4.5, pH 5.4, pH 8.5, and pH 12, respectively. Furthermore, compared to pH 4.5 and pH 5.4, the AgNPs generated at pH 8.5 had a narrower size distribution (Figure 14). This can be explained by the fact that in an acidic medium with a higher concentration of H^+^, the reduction process of Ag^+^ to Ag^0^ is less favorable for the creation of tiny AgNPs.

Furthermore, Table 2 shows that the λ_max_ value peaked at around 456 nm for acidic conditions (pH 4.5) and blue-shifted toward a shorter wavelength of approximately 420 nm for alkaline conditions (pH 8.5). This shift suggests that the size of synthesized AgNPs tends to decrease as the pH value increases. The rapid nucleation process in alkaline conditions produces a large number of small particles, resulting in smaller AgNPs. Conversely, the sluggish nucleation rate at acidic pH results in the formation of fewer, larger particles. This phenomenon has been observed by other research teams as well [55]. The λ_max_ did not significantly change when comparing pH values of 8.5 and 12; however, the absorbance band’s FWHM was broader at pH 12 than at pH 8.5, suggesting a wider particle size dispersion at the higher pH. As a result, it was determined that the ideal pH for the reaction medium was 8.5.

Moreover, Sun et al. [56] found that chitosan chains break in an acidic aqueous solution, which may lessen chitosan’s ability to stabilize metallic particles to some extent. A number of investigations have been conducted recently on the γ-irradiation method of generating AgNPs in chitosan solution [57,58,59]; however, the impact of pH has not yet been examined. Nonetheless, research has been carried out on how pH affects other stabilizers. For example, pH 12.4 was shown to be the optimal value for AgNPs production in carboxyl methyl chitosan solution by Huang et al. [60]. Neutral and acidic media (pH 2–4) were shown to be favored for the production of Ag clusters on SiO_2_, according to Ramnani et al. [61].

As a result, pH is crucial for the production of small AgNPs, and the ideal pH range can change based on the stabilizer agents employed. According to our findings, the synthesis of small AgNPs is best suited for a slightly alkaline medium (pH 8.5).

**Figure 15:**
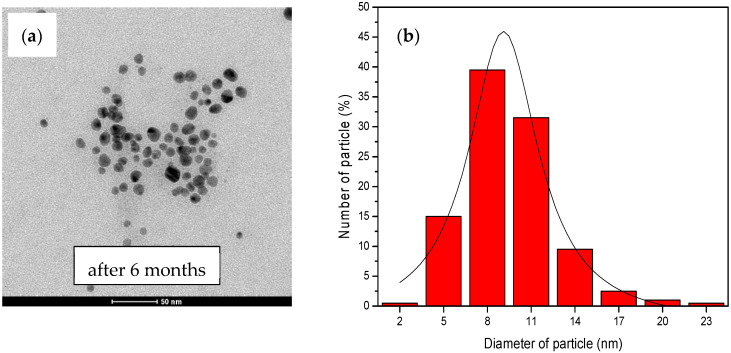
The analysis results from TEM: (**a**) An image of the colloid AgNPs generated from water hyacinth leaf extract after 6 months of UV-A light exposure; (**b**) a histogram of corresponding AgNP particle size distribution.

After storing the AgNPs for 6 months, we found that their shape and size remained spherical, with an average diameter of 10.20 ± 0.21 nm, as shown in Figure 15. These measurements were consistent with the original experimental results, demonstrating the excellent performance and stability of the AgNPs over a long period of time. The special properties of the AgNPs were verified using TEM with our current technique.

Overall, the results suggest that pH level and the addition of capping agents such as starch can control the size and quantity of AgNPs synthesized from water hyacinth leaf extract. These findings have practical implications for advancing the green and sustainable synthesis of AgNPs with desired characteristics.

## 4. Conclusions and Future Directions

This research presents valuable insights into the optimal conditions for synthesizing AgNPs from water hyacinth extract, outlining potential future applications and research directions. The conclusions indicate that the optimal conditions for synthesizing AgNPs involve adjusting the pH level of the AgNP solution to 8.5, adding starch as a stabilizer, exposing the mixture to UV-A radiation, and setting the reaction time to one hour. The experimental findings showed that these conditions produced AgNPs with an average size of 10.60 ± 0.19 nm, with a synthesis yield as high as 99.87%.

The highlight of this research is the use of UV light as a control in the AgNP synthesis process, which offers several benefits: (1) controllable particle size and distribution; (2) reduced energy consumption; and (3) faster response time. The experimental results demonstrated that UV light significantly accelerates the AgNP synthesis reaction, completing the process in just one hour and reducing the synthesis time by 3–50 times compared to traditional methods. Consequently, we chose UV light as a catalyst instead of heat due to its easier control and greater efficiency. Additionally, the AgNPs colloids remain stable and effective even after several months. The special properties of AgNPs indicate their potential for future applications. We have explored their use as antibacterial agents and sensors for uric acid detection in our upcoming research.

Furthermore, the research highlights the potential of water hyacinth leaf extract as a viable and sustainable source for synthesizing AgNPs. Various primary and secondary metabolites in water hyacinth extract are suggested to contribute to the reduction of Ag^+^ ions to Ag^0^, with the reduction rate increasing upon exposure to UV irradiation. This environmentally friendly and rapid synthesis method offers promise for addressing challenges related to aquatic weed management and water pollution. 

The future directions outlined in this section emphasize the potential applications of the synthesized AgNPs, including their use as sensors for uric acid detection in human serum. This involves a new colorimetric sensor based on AgNPs for detecting uric acid. The AgNP–uricase sensor offers the benefits of a quick assay without the need for sample pretreatment and uses inexpensive equipment. Future applications of this sensor will focus on detecting uric acid in human serum, indicating a promising future for clinical diagnostics. Moreover, this work paves the way for the development of innovative biomarker-sensing techniques. Current studies are ongoing, and the results are expected to be presented soon. 

In addition to the above, various types of metal nanoparticles, such as silver, gold, mixed nanomaterials, and nanocomposite materials, can serve as references for the design and synthesis of different metal nanoparticles. In the future, we may explore their applications in energy and sensor technologies more deeply, focusing on sustainable and environmentally friendly energy storage systems [62,63]. This expanded scope of research promises to enhance our understanding and development of advanced materials for various technological applications. 

## Figures and Tables

**Table 1:** λ_max_, FWHM, and average sizes of AgNPs synthesized from water hyacinth leaf extract exposed to different types of UV radiations.

UV	λ_max_ (nm)	FWHM (nm)	Average Size (nm)
UV-A	430	137.73	12.54 ± 0.19
UV-B	440	143.06	13.14 ± 0.23
UV-C	500	173.54	18.39 ± 0.48

**Table 2:** The amount of AgNPs extracted from water hyacinth leaves under UV-A irradiation conditions for 1 h at different pH levels.

Sample	λ_max_ (nm)	Average Size (nm)	Intensity (a.u.)	Yield (%)
UV-A pH 4.5 (starch)	456	25.25 ± 0.70	1.01	86.92
UV-A pH 5.4 (starch)	441	24.74 ± 0.71	1.72	95.61
UV-A pH 8.5 (starch)	420	10.60 ± 0.19	2.66	99.87
UV-A pH 12 (starch)	430	12.56 ± 0.42	2.17	96.07

## Data Availability

The original contributions presented in the study are included in the article, further inquiries can be directed to the corresponding author/s.

## References

[1] Khan I., Saeed K., Khan I. (2019). Nanoparticles: Properties, applications and toxicities. Arab. J. Chem..

[2] Murthy S.K. (2007). Nanoparticles in modern medicine: State of the art and future challenges. Int. J. Nanomed..

[3] Sharma A., Agarwal P., Sebghatollahi Z., Mahato N. (2023). Functional Nanostructured Materials in the Cosmetics Industry: A Review. ChemEngineering.

[4] Wong Y.W.H., Yuen C.W.M., Leung M.Y.S., Ku S.K.A., Lam H.L.I. (2006). Selected applications of nanotechnology in textiles. Autex Res. J..

[5] Villalba-Rodríguez A.M., Martínez-Zamudio L.Y., Hernández Martínez S.A., Rodríguez-Hernández J.A., Melchor-Martínez E.M., Flores-Contreras E.A., González-González R.B., Parra-Saldívar R. (2023). Nanomaterial Constructs for Catalytic Applications in Biomedicine: Nanobiocatalysts and Nanozymes. Top. Catal..

[6] Asghar N., Hussain A., Nguyen D.A., Ali S., Hussain I., Junejo A., Ali A. (2024). Advancement in nanomaterials for environmental pollutants remediation: A systematic review on bibliometrics analysis, material types, synthesis pathways, and related mechanisms. J. Nanobiotechnol..

[7] Gupta D., Boora A., Thakur A., Gupta T.K. (2023). Green and sustainable synthesis of nanomaterials: Recent advancements and limitations. Environ. Res..

[8] Lee S.H., Jun B.H. (2019). Silver Nanoparticles: Synthesis and Application for Nanomedicine. Int. J. Mol. Sci..

[9] Alshehri A.H., Jakubowska M., Młożniak A., Horaczek M., Rudka D., Free C., Carey J.D. (2012). Enhanced Electrical Conductivity of Silver Nanoparticles for High Frequency Electronic Applications. ACS Appl. Mater. Interfaces.

[10] Nguyen N.P.U., Dang N.T., Doan L., Nguyen T.T.H. (2023). Synthesis of Silver Nanoparticles: From Conventional to ‘Modern’ Methods—A Review. Processes.

[11] Zhang X.F., Liu Z.G., Shen W., Gurunathan S. (2016). Silver Nanoparticles: Synthesis, Characterization, Properties, Applications, and Therapeutic Approaches. Int. J. Mol. Sci..

[12] Alharbi N.S., Alsubhi N.S., Felimban A.I. (2022). Green synthesis of silver nanoparticles using medicinal plants: Characterization and application. J. Radiat. Res. Appl. Sci..

[13] Nair L.S., Laurencin C.T. (2007). Silver Nanoparticles: Synthesis and Therapeutic Applications. J. Biomed. Nanotechnol..

[14] Vishwanath R., Negi B. (2021). Conventional and green methods of synthesis of silver nanoparticles and their antimicrobial properties. Curr. Res. Green Sustain. Chem..

[15] Rahman H., Rauf A., Khan S.A., Ahmad Z., Alshammari A., Alharbi M., Alam A., Suleria H.A.R. (2023). Green Synthesis of Silver Nanoparticles Using *Rhazya stricta* Decne Extracts and Their Anti-Microbial and Anti-Oxidant Activities. Crystals.

[16] Jorge de Souza T.A., Rosa Souza L.R., Franchi L.P. (2019). Silver nanoparticles: An integrated view of green synthesis methods, transformation in the environment, and toxicity. Ecotoxicol. Environ. Saf..

[17] Iravani S., Korbekandi H., Mirmohammadi S.V., Zolfaghari B. (2014). Synthesis of silver nanoparticles: Chemical, physical and biological methods. Res. Pharm. Sci..

[18] Szczyglewska P., Feliczak-Guzik A., Nowak I. (2023). Nanotechnology–General Aspects: A Chemical Reduction Approach to the Synthesis of Nanoparticles. Molecules.

[19] Dhaka A., Mali S.C., Sharma S., Trivedi R. (2023). A review on biological synthesis of silver nanoparticles and their potential applications. Results Chem..

[20] Li X., Xu H., Chen Z.S., Chen G. (2011). Biosynthesis of Nanoparticles by Microorganisms and Their Applications. J. Nanomater..

[21] Khan F., Shahid A., Zhu H., Wang N., Javed M.R., Ahmad N., Xu J., Alam M.A., Mehmood M.A. (2022). Prospects of algae-based green synthesis of nanoparticles for environmental applications. Chemosphere.

[22] El-Sheekh M.M., Morsi H.H., Hassan L.H.S., Ali S.S. (2022). The efficient role of algae as green factories for nanotechnology and their vital applications. Microbiol. Res..

[23] Filho S.A., dos Santos M.S., dos Santos O.A.L., Backx B.P., Soran M.L., Opriş O., Lung I., Stegarescu A., Bououdina M. (2023). Biosynthesis of Nanoparticles Using Plant Extracts and Essential Oils. Molecules.

[24] Ansari M., Ahmed S., Abbasi A., Khan M.T., Subhan M., Bukhari N.A., Hatamleh A.A., Abdelsalam N.R. (2023). Plant mediated fabrication of silver nanoparticles, process optimization, and impact on tomato plant. Sci. Rep..

[25] Soni V., Raizada P., Singh P., Cuong H.N., Selvasembian R., Saini A., Saini R.V., Le Q.V., Nadda A.K., Le T.T. (2021). Sustainable and green trends in using plant extracts for the synthesis of biogenic metal nanoparticles toward environmental and pharmaceutical advances: A review. Environ. Res..

[26] Bao Y., He J., Song K., Guo J., Zhou X., Liu S. (2021). Plant-Extract-Mediated Synthesis of Metal Nanoparticles. J. Chem..

[27] Ali M.A., Ahmed T., Wu W., Hossain A., Hafeez R., Islam Masum M.M., Wang Y., An Q., Sun G., Li B. (2020). Advancements in Plant and Microbe-Based Synthesis of Metallic Nanoparticles and Their Antimicrobial Activity against Plant Pathogens. Nanomaterials.

[28] Bahrulolum H., Nooraei S., Javanshir N., Tarrahimofrad H., Mirbagheri V.S., Easton A.J., Ahmadian G. (2021). Green synthesis of metal nanoparticles using microorganisms and their application in the agrifood sector. J. Nanobiotechnology.

[29] Castañeda-Aude J.E., Morones-Ramírez J.R., De Haro-Del Río D.A., León-Buitimea A., Barriga-Castro E.D., Escárcega-González C.E. (2023). Ultra-Small Silver Nanoparticles: A Sustainable Green Synthesis Approach for Antibacterial Activity. Antibiotics.

[30] Asif M., Yasmin R., Asif R., Ambreen A., Mustafa M., Umbreen S. (2022). Green Synthesis of Silver Nanoparticles (AgNPs), Structural Characterization, and their Antibacterial Potential. Dose-Response.

[31] Goyal G., Hwang J., Aviral J., Seo Y., Jo Y., Son J., Choi J. (2016). Green synthesis of silver nanoparticles using β-glucan, and their incorporation into doxorubicin-loaded water-in-oil nanoemulsions for antitumor and antibacterial applications. J. Ind. Eng. Chem..

[32] Melkamu W.W., Bitew L.T. (2021). Green synthesis of silver nanoparticles using *Hagenia abyssinica (Bruce) J.F. Gmel* plant leaf extract and their antibacterial and anti-oxidant activities. Heliyon.

[33] Arshad F., Naikoo G.A., Hassan I.U., Chava S.R., El-Tanani M., Aljabali A.A., Tambuwala M.M. (2023). Bioinspired and Green Synthesis of Silver Nanoparticles for Medical Applications: A Green Perspective. Appl. Biochem. Biotechnol..

[34] Banerjee P., Satapathy M., Mukhopahayay A., Das P. (2014). Leaf extract mediated green synthesis of silver nanoparticles from widely available Indian plants: Synthesis, characterization, antimicrobial property and toxicity analysis. Bioresour. Bioprocess..

[35] Habibullah G., Viktorova J., Ulbrich P., Ruml T. (2022). Effect of the physicochemical changes in the antimicrobial durability of green synthesized silver nanoparticles during their long-term storage. RSC Adv..

[36] Bhat R.S., Almusallam J., Daihan S.A., Dbass A.A. (2019). Biosynthesis of silver nanoparticles using Azadirachta indica leaves: Characterisation and impact on Staphylococcus aureus growth and glutathione-S-transferase activity. IET Nanobiotechnol..

[37] Sundeep D., Vijaya Kumar T., Rao P.S.S., Ravikumar R.V.S.S.N., Gopala Krishna A. (2017). Green synthesis and characterization of Ag nanoparticles from *Mangifera indica* leaves for dental restoration and antibacterial applications. Prog. Biomater..

[38] Parameshwaran R., Kalaiselvam S., Jayavel R. (2013). Green synthesis of silver nanoparticles using *Beta vulgaris*: Role of process conditions on size distribution and surface structure. Mater. Chem. Phys..

[39] Ibrahim H.M.M. (2015). Green synthesis and characterization of silver nanoparticles using banana peel extract and their antimicrobial activity against representative microorganisms. J. Radiat. Res. Appl. Sci..

[40] Degaga A.H. (2018). Water Hyacinth (*Eichhornia crassipes*) Biology and its Impacts on Ecosystem, Biodiversity, Economy and Human Well-being. J. Life Sci. Biomed..

[41] Harun I., Pushiri H., Amirul-Aiman A.J., Zulkeflee Z. (2021). Invasive Water Hyacinth: Ecology, Impacts and Prospects for the Rural Economy. Plants.

[42] Mochochoko T., Oluwafemi O.S., Jumbam D.N., Songca S.P. (2013). Green synthesis of silver nanoparticles using cellulose extracted from an aquatic weed; water hyacinth. Carbohydr. Polym..

[43] Hublikar L.V., Ganachari S.V., Raghavendra N., Patil V.B., Banapurmath N.R. (2021). Green synthesis silver nanoparticles via *Eichhornia Crassipes* leaves extract and their applications. Curr. Res. Green Sustain. Chem..

[44] Nandiyanto A.B.D., Ragadhita R., Hoffah S.N., Husaeni D.F.A., Husaeni D.N.A., Fiandini M., Luckiardi S., Soegoto E.S., Darmawan A., Aziz M. (2023). Progress in the utilization of water hyacinth as effective biomass material. Environ. Dev. Sustain..

[45] Su W., Sun Q., Xia M., Wen Z., Yao Z. (2018). The Resource Utilization of Water Hyacinth (*Eichhornia crassipes* [Mart.] Solms) and Its Challenges. Resources.

[46] Oluwafemi O.S., Anyik J.L., Zikalala N.E., Sakho E.H.M. (2019). Biosynthesis of silver nanoparticles from water hyacinth plant leaves extract for colourimetric sensing of heavy metals. Nano-Struct. Nano-Objects.

[47] Munive-Olarte A., Rosano-Ortega G., Schabes-Retchkiman P., Martínez-Gallegos M.S.M., Kassis E.E., González-Pérez M., Pacheco-García F. (2017). Assessment of Biomass of Leaves of Water Hyacinth (Eichhornia crassipes) as Reducing Agents for the Synthesis of Nanoparticles of Gold and Silver. Int. J. Adv. Eng. Manag. Sci..

[48] Kiruba Daniel S.C.G., Nehru K., Sivakumar M. (2012). Rapid Biosynthesis of Silver Nanoparticles using *Eichornia crassipes* and its Antibacterial Activity. Curr. Nanosci..

[49] Martínez-Espinosa J.C., Ramírez-Morales M.A., Carrera-Cerritos R. (2022). Silver Nanoparticles Synthesized Using *Eichhornia crassipes* Extract from Yuriria Lagoon, and the Perspective for Application as Antimicrobial Agent. Crystals.

[50] Thombre R., Chitnis A., Kadam V., Bogawat Y., Colaco R., Kale A. (2014). A facile method for synthesis of biostabilized silver nanoparticles using *Eichhornia crassipes* (Mart.) Solms (water hyacinth). Indian J. Biotechnol..

[51] Raza M.A., Kanwal Z., Rauf A., Sabri A.N., Riaz S., Naseem S. (2016). Size- and Shape-Dependent Antibacterial Studies of Silver Nanoparticles Synthesized by Wet Chemical Routes. Nanomaterials.

[52] Cheon J.Y., Kim S.J., Rhee Y.H., Kwon O.H., Park W.H. (2019). Shape-dependent antimicrobial activities of silver nanoparticles. Int. J. Nanomed..

[53] Morones J.R., Elechiguerra J.L., Camacho A., Holt K., Kouri J.B., Ramírez J.T., Yacaman M.J. (2005). The bactericidal effect of silver nanoparticles. Nanotechnology.

[54] Agnihotri S., Mukherji S., Mukherji S. (2014). Size-controlled silver nanoparticles synthesized over the range 5–100 nm using the same protocol and their antibacterial efficacy. RSC Adv..

[55] Chitra K., Annadurai G. (2014). Antibacterial Activity of pH-Dependent Biosynthesized Silver Nanoparticles against Clinical Pathogen. BioMed Res. Int..

[56] Sun C., Qu R., Chen H., Ji C., Wang C., Sun Y., Wang B. (2008). Degradation behavior of chitosan chains in the ‘green’ synthesis of gold nanoparticles. Carbohydr. Res..

[57] Chen P., Song L., Liu Y., Fang Y. (2007). Synthesis of silver nanoparticles by γ-ray irradiation in acetic water solution containing chitosan. Radiat. Phys. Chem..

[58] Long D., Wu G., Chen S. (2007). Preparation of oligochitosan stabilized silver nanoparticles by gamma irradiation. Radiat. Phys. Chem..

[59] Yoksan R., Chirachanchai S. (2009). Silver nanoparticles dispersing in chitosan solution: Preparation by γ-ray irradiation and their antimicrobial activities. Mater. Chem. Phys..

[60] Huang L., Zhai M.L., Long D.W., Peng J., Xu L., Wu G.Z., Li J.Q., Wei G.S. (2008). UV-induced synthesis, characterization and formation mechanism of silver nanoparticles in alkalic carboxymethylated chitosan solution. J. Nanopart. Res..

[61] Ramnani S.P., Biswal J., Sabharwal S. (2007). Synthesis of silver nanoparticles supported on silica aerogel using gamma radiolysis. Radiat. Phys. Chem..

[62] Huang Q., Guo Y., Chen D., Zhang L., Li T.-T., Hu Y., Qian J., Huang S. (2021). Rational construction of ultrafine noble metals onto carbon nanoribbons with efficient oxygen reduction in practical alkaline fuel cell. Chem. Eng. J..

[63] Chen D., Han C., Sun Q., Ding J., Huang Q., Li T.-T., Hu Y., Qian J., Huang S. (2023). Bimetallic AgNi nanoparticles anchored onto MOF-derived nitrogen-doped carbon nanostrips for efficient hydrogen evolution. Green Energy Environ..

